# Normal histone modifications on the inactive X chromosome in ICF and Rett syndrome cells: implications for methyl-CpG binding proteins

**DOI:** 10.1186/1741-7007-2-21

**Published:** 2004-09-20

**Authors:** Stanley M Gartler, Kartik R Varadarajan, Ping Luo, Theresa K Canfield, Jeff Traynor, Uta Francke, R Scott Hansen

**Affiliations:** 1 Department of Medicine, University of Washington, Seattle, WA, USA; 2 Department of Genome Sciences, University of Washington, Seattle, WA, USA; 3 Department of Genetics, Stanford University, Stanford, CA, USA

## Abstract

**Background:**

In mammals, there is evidence suggesting that methyl-CpG binding proteins may play a significant role in histone modification through their association with modification complexes that can deacetylate and/or methylate nucleosomes in the proximity of methylated DNA. We examined this idea for the X chromosome by studying histone modifications on the X chromosome in normal cells and in cells from patients with ICF syndrome (Immune deficiency, Centromeric region instability, and Facial anomalies syndrome). In normal cells the inactive X has characteristic silencing type histone modification patterns and the CpG islands of genes subject to X inactivation are hypermethylated. In ICF cells, however, genes subject to X inactivation are hypomethylated on the inactive X due to mutations in the DNA methyltransferase (DNMT3B) genes. Therefore, if DNA methylation is upstream of histone modification, the histones on the inactive X in ICF cells should not be modified to a silent form. In addition, we determined whether a specific methyl-CpG binding protein, MeCP2, is necessary for the inactive X histone modification pattern by studying Rett syndrome cells which are deficient in MeCP2 function.

**Results:**

We show here that the inactive X in ICF cells, which appears to be hypomethylated at all CpG islands, exhibits normal histone modification patterns. In addition, in Rett cells with no functional MeCP2 methyl-CpG binding protein, the inactive X also exhibits normal histone modification patterns.

**Conclusions:**

These data suggest that DNA methylation and the associated methyl-DNA binding proteins may not play a critical role in determining histone modification patterns on the mammalian inactive X chromosome at the sites analyzed.

## Background

Although it has been known for some time that histone modifications play a role in gene expression [1], it is only in the last several years that the details of these modifications have been more fully described. Acetylation and methylation of histone tails, for example, exhibit characteristic patterns for expressed and repressed genes in all eukaryotes studied [2]. This generality of histone modification and gene expression holds for eukaryotes with and without DNA methylation, indicating that DNA methylation is not required for histone modification. In organisms with DNA methylation, however, interactions between histone modification and DNA methylation do appear to exist.

In *Neurospora*, histone methylation appears to determine DNA methylation patterns [3,4]. In *Arabidopsis*, non-CpG DNA methylation also appears to be determined by histone methyltransferases, whereas CpG methylation does not [5,6]. In mammals, there is considerable evidence suggesting that methyl-CpG binding proteins may play a significant role in histone modification through their association with histone deacetylases [7-11]. Mutations in the MeCP2 methyl-DNA binding protein, which are the cause of most Rett syndrome cases [12], support this model, because human male and female cells with *MECP2 *mutations exhibit histone hyperacetylation [10]. Histone hyperacetylation was also observed in mice with *Mecp2 *mutations [13]. Thus, DNA methylation is upstream of histone modification in this model of methyl-DNA binding proteins and histone modification. Another possibility is that DNA methyltransferases themselves may target histone deacetylases through a noncatalytic domain, leading to histone modifications that are independent of other methyl-DNA binding proteins [14].

We are especially interested in the X chromosome with respect to the question of the relationship between DNA methylation and histone modification. The mammalian X chromosome is unusual in that about a thousand gene-associated CpG islands are hypermethylated on the inactive X and hypomethylated on the active X. Except for imprinted loci, methylation patterns at most other regions of the genome are similar between homologs. Histone modification differences known to be associated with either silent or expressed chromatin also distinguish the active and inactive X chromosomes [15-19]. Thus, the mammalian X chromosome inactivation system would appear ideal for testing whether or not a methyl DNA binding protein – histone modification pathway exists for the inactive X chromosome.

To examine more fully the possible relationships between DNA methylation and histone modification, we have utilized cell cultures from individuals with a human hypomethylation disease called the ICF syndrome. This disease is clinically characterized by "Immune deficiency, Centromeric region instability, and Facial anomalies". In most cases, the molecular defects result from mutations in the DNMT3B methyltransferase gene [20-22]. Certain heterochromatic regions are markedly hypomethylated as a result of these mutations, including the CpG islands on the inactive X chromosome that are associated with genes [23] and LINE-1 elements [24]. If DNA methylation is upstream of histone modification, the histones on the inactive X should not be modified to a silent form in ICF cells. Our results indicate, however, that these histones do have modifications typical of silenced genes, suggesting that methyl-DNA binding proteins may not be critical with respect to histone modification on the inactive X chromosome. In addition, we examined clonal primary fibroblast cultures from two individuals with Rett syndrome and found that the histone modification pattern of the inactive X is not affected by mutations in *MECP2*. This suggests that this specific methyl-DNA binding protein does not have a major role in silencing the inactive X through histone modification.

## Results

### Cytological analysis of histone modification

#### DNMT3B mutant cells (ICF syndrome)

**Figure 1:**
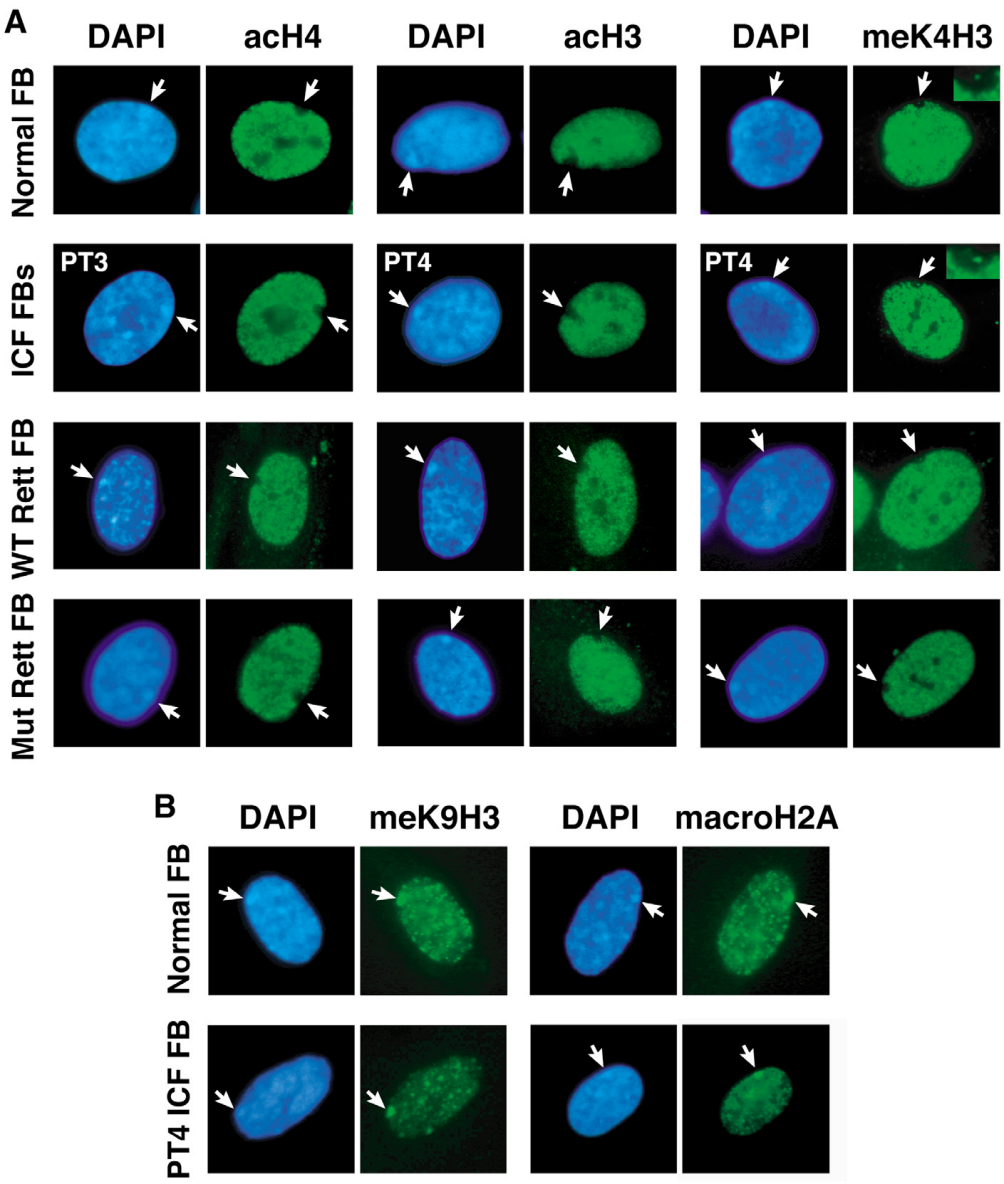
**The interphase inactive X in normal and mutant cells: histone modification and macroH2A1 association. **Photomicrograph examples of normal, ICF, and Rett fibroblasts that were FITC-labeled using antisera to various modified histones. Arrows point to sex chromatin on DAPI-stained cells, and to the corresponding sex chromatin site in the FITC-labeled photo. A. Normal, ICF, and Rett fibroblasts FITC-labeled using antisera to acetylated histone H4 (acH4), acetylated histone H3 (acH3), and dimethylated K4 histone H3 (meK4H3). Note that the sex chromatin body is not stained by these antibodies and appears as a hole or a gap that occasionally contains a prominent dot (see insets). This FITC-stained dot appears to correspond to the DXZ4 domain, as described in the text. B. Normal and ICF fibroblasts labeled with antibody to dimethylated K9 histone H3 (meK9H3) and macrohistone H2A1 (macroH2A).

**Figure 2:**
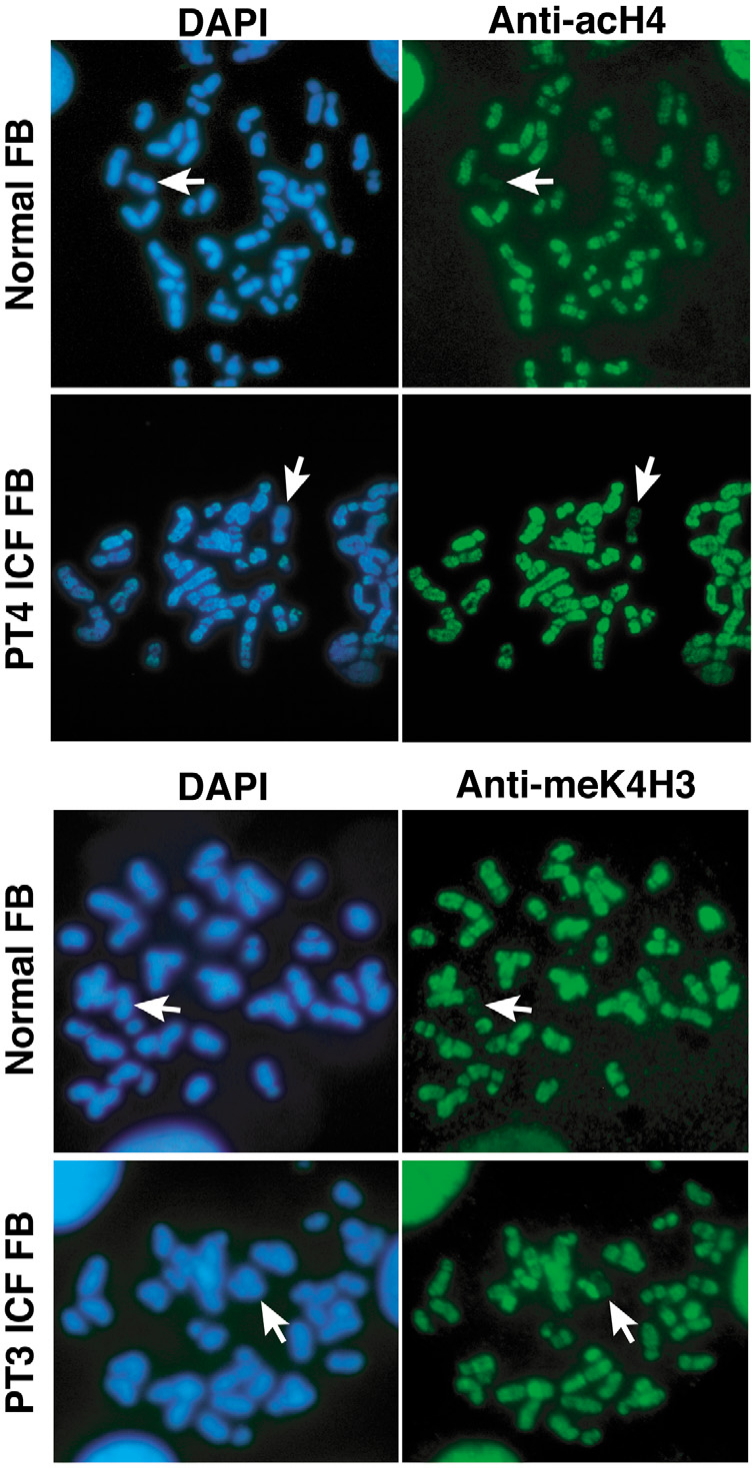
**Histone modification on the inactive X in metaphases from normal and ICF fibroblasts (FB). **Examples are shown of normal and ICF (PT3 and PT4) metaphase cells labeled with antisera to acetylated histone H4 (anti-acH4) and dimethylated K4 histone H3 (anti-meK4H3). Arrows point to X chromosomes stained with DAPI that correspond to Xs stained lightly with FITC-antibodies. Note the densely-stained acH4 region on the tip of the inactive X short arm in PT4. Such staining is also seen in normal cells and corresponds to the actively transcribed pseudoautosomal region. These observations indicate that on the inactive X, H4 is hypoacetylated and K4 H3 is hypomethylated in both normal and ICF cells.

We examined histone modifications known to be associated with the inactive X chromosome in two ICF cell lines and normal control cells. Specific histone modifications including histone H3 and H4 acetylation, and histone H3 methylation at K4 and K9, were examined using antibodies to stain nuclei and metaphases [15-17]. We also examined histone macroH2A1 staining, which is known to be concentrated on the inactive X at interphase [25]. One hundred or more interphase nuclei that had an obvious sex chromatin body by DAPI staining were scored for histone modification. For acetylated histone (H3 and H4) and K4-methylated histone H3, the majority (>60%) of nuclei showed a characteristic hole at the sex chromatin body in both normal and ICF cells (Fig. 1A). For histone H3 methylation at K9, the frequency of positive cells (Fig. 1B) was lower (about 50%). We often noted a positive signal for methylated K4 histone H3 in an otherwise negative-staining sex chromatin region in both normal and ICF cells (Fig. 1A). This signal appears to derive from the DXZ4 locus that was previously reported by Chadwick and Willard [19] as having active-type histone modifications. DXZ4 is a megabase-sized region known to be hypermethylated on the active X and hypomethylated on the inactive X in normal cells [26]; this locus appears to be modified normally in ICF cells. Surprisingly, we did not observe this signal on metaphase spreads, suggesting that our resolution on these preparations may be much lower than on interphase chromatin. For metaphase spreads (acetylated histone H3 and H4 and histone H3 methylated at K4) we analyzed 50 cells per line, and in the great majority of analyzable metaphases (>80%) a single lightly-labeled chromosome was detected (Fig. 2). In some of these cells, the tip of the short arm was labeled, as would be expected for the pseudoautosomal region (Fig. 2).

The expected patterns of macroH2A1 histone concentration and histone modification on the inactive X were found in cells derived from ICF individuals and in control normal cells (Figs. 1 and 2 and Table 1). At the cytological level, therefore, no difference could be found between normal and ICF cells with respect to the histone modifications on the inactive X.

#### MECP2 mutant cells (Rett syndrome)

We examined histone modifications and macrohistone association in clones from two individuals heterozygous for a mutation in *MECP2*, an X-linked gene that is subject to X inactivation [12]. *MECP2 *mutations lead to Rett syndrome, and the protein product codes for a methyl-CpG binding protein known to recruit a transcriptional silencing complex that deacetylates histones. In Rett syndrome and in mice with mutant *Mecp2*, histones exhibit hyperacetylation [10,13], as would be expected if this methyl-DNA binding protein functions upstream of histone modification. In one case, we had complementary clones with either the mutant (1786YY) or wild type *MECP2 *allele (1786QQ) on the inactive X, and in the second case, we had one clone with the wild type *MECP2 *on the inactive X (1789V) [27]. For all the histone modifications we examined (H3 and H4 acetylation and H3 K4 and K9 methylation), the cytological patterns on the active and inactive X chromosomes in mutant MeCP2-expressing cells were indistinguishable from those in clones expressing the wild type allele or in other control cultures (Fig. 1). These results suggest that MeCP2 does not function in determining these histone modification patterns on the inactive X chromosome.

### ChIP analysis

To verify our cytological histone modification results at the gene level, we searched for promoter polymorphisms at seven X-linked loci (*G6PD*, *NEMO*, *MECP2*, *SYBL1*, *AR*, *FMR1*, and *PGK1*) in ICF cells so that we could employ allele-specific chromatin immunoprecipitation (ChIP) analysis. We restricted our search for polymorphisms to the promoter region, as several reports have indicated that marked differences in histone modifications between active and inactive alleles are seldom detected at other regions [28-30]. We found useful promoter polymorphisms at two loci, *SYBL1 *(synaptobrevin-like gene in the pseudoautosomal portion of Xq28) and *AR *(androgen receptor in Xq12).

**Figure 3:**
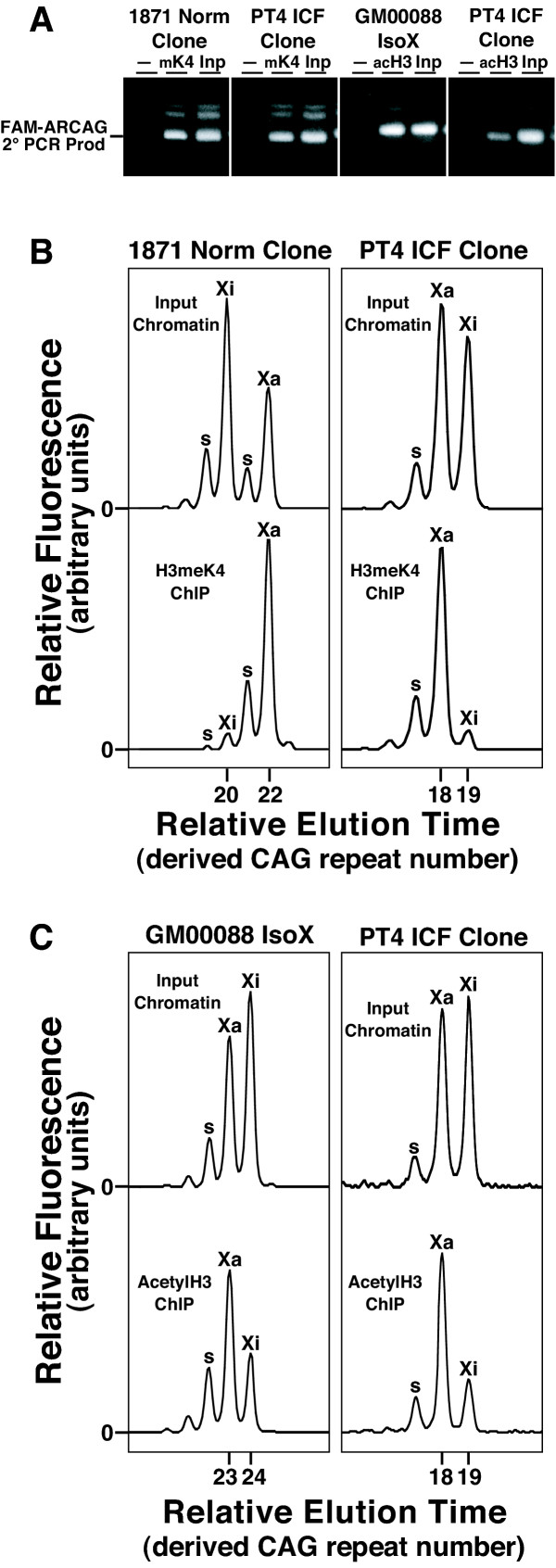
**ChIP analysis of histone modification at the promoter region of the *AR *gene. **Allele-specific ChIP analysis for H3 acetylation and H3 K4 methylation was performed using the highly polymorphic CAG repeat region in the 5' coding region of the androgen receptor in Xq12. A. "Normal" isoX fibroblasts with highly skewed inactivation (GM00088) and cloned fibroblasts from normal (1871) and ICF (PT4) cultures were subjected to ChIP and the immunoprecipitated material was amplified for *AR *analysis in two stages; nested FAM-labeled primers were used in the second stage for size analysis on an automated sequencer. The ethidium bromide-stained agarose gel of the secondary PCR products show strong signals in the input (Inp), anti-H3 dimethyl K4 antibody (mK4), and anti-H3 acetyl K9, K14 (acH3), but little or no signal in the "no antibody" controls (-). The weak bands with slower migration correspond to heteroduplexes with the primary amplimer, but these do not interfere with the allele-specific analysis of the denatured product. B. and C. Allele-specific analyses of the products shown in A, using an automated sequencer, revealed that the active X alleles (Xa) in normal cells (1871 and GM00088) are hypermethylated at H3 K4 (H3meK4) and hyperacetylated at H3 K9, K14 (AcetylH3), whereas the corresponding inactive X (Xi) alleles are hypomethylated and hypoacetylated. ICF cells (PT4) showed the same active X versus inactive X modifications as normal cells. "Shadow bands" (s) probably correspond to PCR errors. Allele assignment was ascertained by a similar method using allele-specific RT-PCR with DNased RNA.

Previously, one of us (RSH) has reported on a ChIP study at the *SYBL1 *locus in male ICF cells where the inactive Y allele had reactivated and the histone modification pattern was that of an active gene [31]. Here we report on ChIP studies at the *SYBL1 *and *AR *loci in ICF female cultures using antibodies to histone H3 dimethylated at K4 and to acetylated histone H3. Both loci are subject to X inactivation, and the inactive X alleles remain inactive in ICF cells even though the 5' CpG islands are hypomethylated [23]. In the case of the *SYBL1 *inactive X allele, the methylation level is reduced by over 90% with most chromosomes exhibiting no methylation. An XhoI restriction site polymorphism in the untranslated exon 1 of *SYBL1 *permitted separation of the active and inactive alleles in cloned cells. A CAG repeat number polymorphism in the 5' coding region of the *AR *gene (1.3 kb downstream of the transcription start site according to reference sequence NM000044) was informative in one ICF sample (PT 4) and in several controls, thus permitting separation of the active and inactive alleles in cloned cells, and in cultures with highly skewed X inactivation. The antibodies were highly specific under the amplification conditions chosen, so that a strong signal was seen for the pull-down experiment with antibody and little or no signal for the "no antibody" control (Fig. 3A).

The fluorescent amplification products from the *AR *gene were then separated on an automated sequencer according to CAG repeat number. Two major peaks are detected in the input control DNA, corresponding to the active (A) and inactive (I) X alleles, differing in CAG repeat number (Fig. 3B). An allele was determined to be from the active X by RT-PCR analysis (data not shown). The lesser "shadow band" peaks, labeled S, probably derive from PCR errors. In the methylated K4 H3 and acetylated H3 antibody ChIPs, a single peak predominates in both normal and ICF cells (Figs. 3B and 3C) that corresponds to the active X allele.

**Figure 4:**
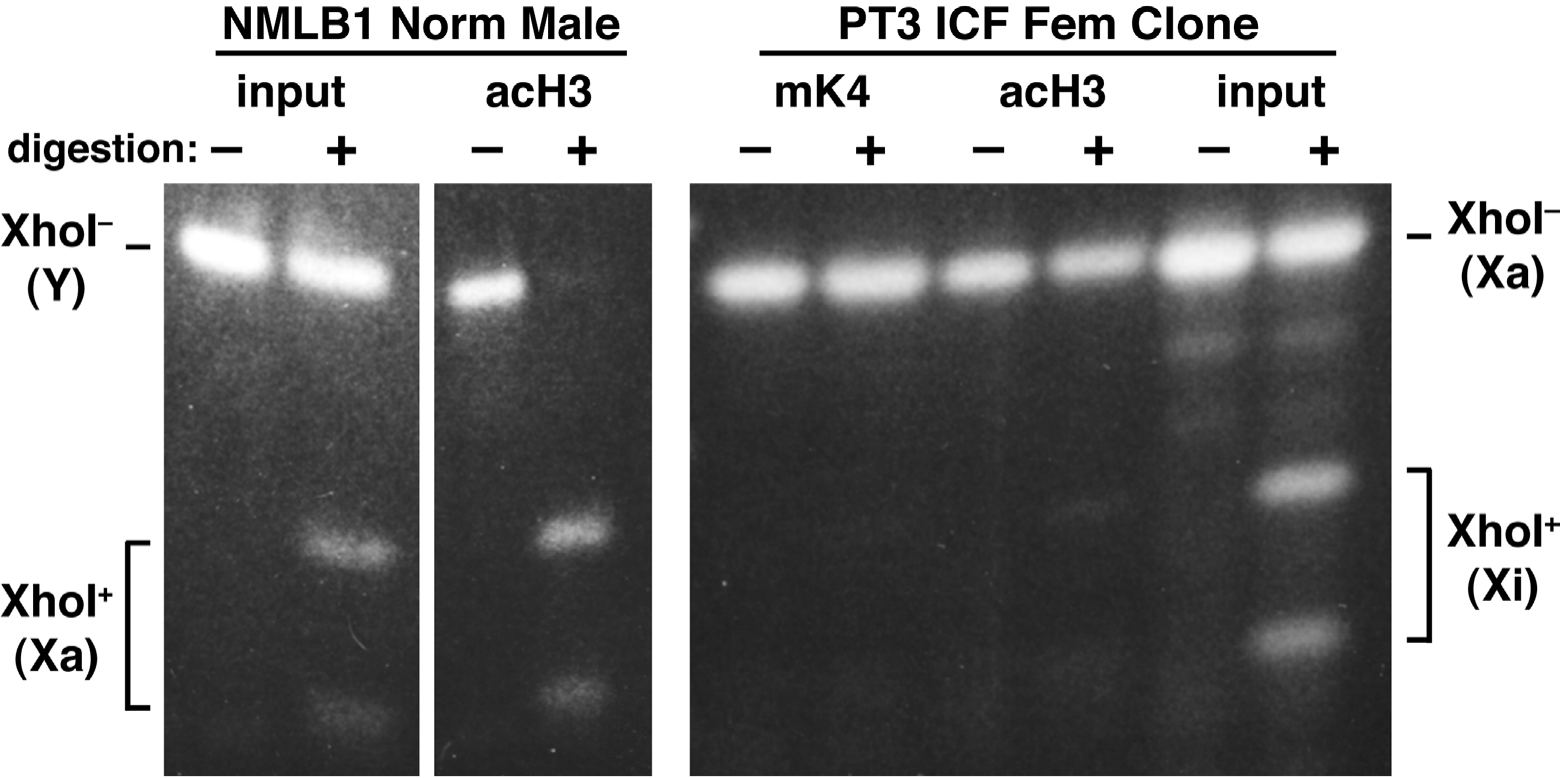
**ChIP analysis of histone modification at the SYBL1 promoter region. **Allele-specific ChIP analysis for H3 acetylation and H3 K4 methylation was performed using an XhoI polymorphism in the 5' UTR of the *SYBL1 *gene in the Xq28 pseudoautosomal region that is also present on the Y chromosome. In normal male cells, the Y-linked locus is inactivated, hypermethylated, and late-replicating as is the inactive X allele in female cells [23,31,49]. To determine if this region has abnormal histone modifications on the X-inactivated but DNA hypomethylated ICF female X, ChIP assays were performed for acetylated histone H3 (acH3) and K4-methylated histone H3 (mK4) in normal male lymphoblasts and in PT3 ICF female fibroblasts; the ethidium bromide-stained gels of each sample are shown before and after digestion with XhoI. The undigested alleles (XhoI+) are 268 bp; the digested alleles (XhoI-) result in fragments of 108 and 260 bp. The ChIP assay for acetylated histone H3 (acH3) shows that only the XhoI-digested allele (XhoI+) is hyperacetylated in a normal male lymphoblast (NMLB1), and this corresponds to the active X allele (Xa) by RT-PCR (data not shown). An hTERT-transformed clone of PT3 ICF fibroblasts was also analyzed by ChIP. This clone has normal monoallelic expression of *SYBL1 *even though the promoter region is extremely hypomethylated as determined by bisulfite methylation analysis of DNA. The inactive X allele in the PT3 clone is hypoacetylated at histone H3 and hypomethylated at H3K4 because only the active X allele (XhoI-) is immunoprecipitated with either the acetylated or K4-methylated histone H3 antibodies (although a small portion of the inactive X also appears to have been precipitated by the acetylated H3 antibody).

Our ChIP analysis of the inactive X at the *SYBL1 *locus in an ICF female (PT3) also showed normal histone H3 hypoacetylation and K4 H3 hypomethylation in spite of the very low levels of DNA methylation in this CpG island region (Fig. 4). These data, therefore, agree with our cytological observations in that only the active X alleles are positive for the histone modifications known to be associated with active genes, though a minor portion of the inactive X allele was found to precipitate with the acetylated H3 antibody in both normal and ICF cells (Fig. 3C and data not shown).

## Discussion

The major observation reported here is that ICF cells, despite being hypomethylated at gene- and L1-associated CpG islands on the inactive X chromosome, exhibit the same histone modification patterns as inactive Xs in normal cells. In addition, we show that cells mutant for MeCP2, a methyl DNA binding protein, also exhibit normal histone modification patterns on the inactive X. These results imply that DNA methylation and/or this methyl DNA binding protein are not critical for determining histone modification patterns on the inactive X chromosomes.

Two major questions can be raised about our results: (1) is the sensitivity of the cytological histone modification assay too low to detect active-type histone modifications on the ICF inactive X? and (2) is the extent of methylation on the ICF inactive X greater than is suggested by CpG island and LINE-1 methylation patterns?

The cytological results imply that most of the genes on the inactive X in ICF cells are subject to inactivation, a conclusion supported by our allele-specific expression analyses of individual genes, such as *AR*, in ICF cells ([23] and data not shown). For genes subject to X inactivation in ICF cells, we expect histone modifications at the gene level to be similar to those detected cytologically at the chromosome level, and this is what we have shown here for the *AR *gene. For genes that escape X inactivation in ICF cells, we expect their histone modification patterns to be those of expressed genes, and one of us (RSH) has previously reported this to be the case for the *SYBL1 *gene ([31] and data not shown). We did not detect these active patterns cytologically, suggesting that there are no large blocks of genes escaping inactivation in ICF cells except at the Xp pseudoautosomal region, which normally contains escaping genes (Fig. 2).

Methylation levels at inactive X-linked CpG islands in ICF cells are decreased by an average of 89% from normal as determined by bisulfite analyses at the *G6PD*, *FMR1*, and *SYBL1 *loci, and many of the cloned alleles analyzed were completely unmethylated like active X alleles [23]. It is possible that DNA methylation at other CpG-rich regions (e.g., Alu and LINE-1 elements) on the X chromosome might be differentially methylated and play a role in the X chromosome histone modification pathway. One of us (RSH) has recently shown that LINE-1 elements are hypermethylated on both active and inactive X chromosomes in normal cells but, surprisingly, they are hypomethylated on the inactive X and hypermethylated on the active X in ICF cells [24]. These results argue against a role for LINE-1 methylation in histone modification on the inactive X chromosome. A more complete DNA methylation analysis of the ICF and normal inactive Xs needs to be done, however, because other widespread sequences may be hypermethylated on the ICF inactive X that could direct histone modifications.

Because we know that promoter methylation is important in gene expression, it seems reasonable that if DNA methylation were directly involved in the histone modification pathway, CpG island methylation would play a critical role. Further support for this idea comes from the fact that histone modifications distinguishing active and inactive X-linked genes are concentrated in promoter regions [28,32]. In fact, Rougeulle et al. [32] propose that the promoter-restricted histone modification seen at X-linked loci may be unique to monoallelically-expressed genes and provide them with an epigenetic mark.

That DNA methylation is not critical to the developmental appearance of histone modifications is further supported by recent murine studies showing that differential histone modification of the Xs in early development precede differential developmental appearance of DNA methylation [33,34]. The fact that DNA methylation does not appear to be critical to the development of histone modifications in X-linked gene expression should not be confused with a more important role for DNA methylation in maintaining repression of X-linked genes. Some years ago we showed that the earliest events in reactivating inactive X-linked genes were hemidemethylation followed by chromatin hypersensitivity, and then transcription factor binding and transcription [35,36]. More extensive studies have recently pointed to a similar conclusion [11,37,38]. Thus, DNA methylation appears to play a dominant role in maintaining repression, even though it is a late event in establishing silent chromatin.

We can also consider the implication of this work for the proposed role of methyl-CpG binding proteins in the histone modification pathway. Our ICF cell data and the results from murine developmental studies, showing that histone modification of X-linked genes precedes DNA methylation, argue against such a role for the X chromosome. A role for methyl-DNA binding proteins in the histone modification pathway is supported by studies with Rett syndrome cells where a methyl-DNA binding protein, MeCP2, is mutated. In both humans and mice with Rett syndrome mutations, general hyperacetylation of histones was reported, albeit at different sites. In human cell lines, H4 was hyperacetylated preferentially at K16 [10], while in mouse mutant tissues hyperacetylation was reported specifically at H3K9 [10,13]. In our work, however, we saw no major effect of two different *MECP2 *mutations on inactive X histone modification. The recent discovery that LINE-1 elements on the inactive X are methylated by a methyltransferase distinct from the one that carries out the same modification on the active X raises the possibility that the inactive X could have its own modification rules [24]. We must consider, therefore, the possibility that the inactive X chromosome does not utilize methyl-DNA binding proteins in the histone modification pathway. Such a possibility would fit with the failure to detect protein footprints at promoters on the inactive X chromosome, whereas they are readily detectable on the active X [36,39-42]. It should be noted that only a small fraction of possible histone modifications have been elucidated at this time, and it is possible that histone modification on the inactive X that depends on methyl-DNA binding protein(s) will be found in the future.

Finally, we would like to comment on the implication of this study regarding the inactive X silencing complex. Systems controlling gene expression tend to be multilayered and the X inactivation system is no exception. We know that silencing on the inactive X involves XIST RNA, DNA methylation, histone modification patterns, chromatin sensitivity, and delayed replication. It is our opinion that these factors tend to act in a more or less independent manner, as we have suggested several times in the past [23,43-46]. For example, promoter demethylation of inactive X-linked genes, as occurs in ICF cases, does not necessarily lead to reactivation; markedly advanced replication time must also be present for reactivation to take place [23]. The present study would appear to add further support to this idea.

## Conclusions

The inactive X chromosome in mammalian cells is characterized by a particular set of histone modifications. It has been suggested that methyl-DNA binding proteins may be involved in these modifications through their interactions with histone deacetylases. We have investigated this idea by studying histone modification patterns on the inactive X in ICF and Rett syndrome cells. ICF cells are hypomethylated on the inactive X, in contrast to normal cells, and the Rett cells we studied were deficient in MeCP2, a specific X-linked methyl-DNA binding protein. We found that the histone modification patterns on the inactive X in these mutant cells were indistinguishable from those in normal cells. We conclude that DNA methylation and the associated methyl-DNA binding proteins do not appear to play a critical role in determining histone modification patterns on the mammalian inactive X chromosome, either globally or at the level of the promoter.

## Methods

### Cells and cell culture

Mutant fibroblast cell cultures included two from female ICF individuals, whose *DNMT3B *mutations have been previously described [20], and complementary clonal cultures from an individual (Rett 1) heterozygous for a mutation (1155del132) in the *MECP2 *gene [27]. In one clone (1786YY), the mutant gene is on the active X, leading to a culture with nonfunctional MeCP2 protein; the complementary control clone (1786QQ) has functional MeCP2 because the mutant gene is on the inactive X. In another clone (1789V), derived from a Rett individual with the mutation R106W (Rett 2), the active X carried the mutant allele. Normal fibroblast cultures were obtained from commercial sources. For chromatin precipitation studies, the ICF fibroblast clones were immortalized with hTERT, as previously described [47].

Cells were grown in AmnioMax-C100 (Gibco Invitrogen Corp.; Carlsbad, CA) and harvested in trypsin:EDTA (Gibco Invitrogen Corp.) under standard conditions [46,48].

### Cytology

For analysis of interphase stages, cells were plated on alcohol-washed 22 mm square cover slips in 35 × 10 mm Petri dishes. On the following day the medium was removed and the cells were washed once with PBS followed by fixation in 95% ethanol:5% acetic acid for 1 min at room temperature. The rest of the procedure followed the "Immunocytochemistry Protocol" of Upstate (Lake Placid, NY). DAPI-stained slides mounted in antifade were examined with a Nikon Microphot FXA microscope and images were captured with a Nikon Coolpix 995 digital camera. The inactive X was recognized under DAPI staining as sex chromatin. Absence of a particular histone modification on the inactive X was seen as a hole or gap at the sex chromatin location.

For analysis of metaphase cells, we plated cells in 150 × 25 mm tissue culture dishes and added colcemid (Gibco Invitrogen Corp.) 48 h later (0.1 μg/ml for 2 h). The medium was removed and the cells were washed once with Hanks' balanced salt solution, followed by trypsinization (Gibco Invitrogen Corp.) with slight agitation to collect metaphase cells. Serum was added to stop tryptic action and the cells were recovered by centrifugation, then placed in hypotonic solution (3 mg/ml KCl and 1 mg/ml sodium citrate) at 37°C for 15 min. Cells were collected on to slides using a Cytospin centrifuge and then fixed in 95% ethanol:5% acetic acid for 1 min. The rest of the procedure followed the Upstate protocol mentioned above, followed by DAPI staining, mounting in antifade, and examination with a Nikon Microphot FXA microscope.

Antibodies used to detect histone modifications were obtained from Upstate and included: "Anti-acetyl-Histone H4," recognizing acetylated lysines 5, 8, 12, and 16, "Anti-acetyl-Histone H3 (Lys 9)," "Anti-dimethyl-Histone H3 (Lys 9)," "Anti-dimethyl-Histone H3 (Lys 4)," and "Anti-Histone macroH2A1."

### ChIP studies

Chromatin immunoprecipitation was performed using the protocol of Upstate with slight modifications. For each experiment, a near-confluent 75 cm^2 ^tissue culture flask (about 3 × 10^6 ^cells) was washed with PBS and treated with 4% formaldehyde (pH > 7) for 10 min at 37°C. Protease inhibitor cocktail (Complete) from Roche Diagnostics (Indianapolis, IN) was used in place of individual inhibitors, and Protein A-Separose 4B (Zymed Laboratories Inc.; South San Francisco, CA) was used to collect immune complexes. After elution of immune complexes, they were heated at 65°C for 4 h to reverse crosslinks, and the DNA was recovered with a "QIAquick" PCR purification kit from Qiagen Inc. (Valencia, CA).

Primary amplification of *AR *DNA was across the polymorphic 5' CAG repeat region as previously described [23], using 27–35 cycles of PCR amplification with 10% of the immunoprecipitated material or 50 ng of input DNA in a 50 μl reaction volume. Allele-specific analysis was performed by amplifying the primary product with a 5'-FAM-labeled nested primer as previously described [23], using 6–15 cycles of PCR amplification with 2–10 μl primary product in a 50 μl reaction volume. All amplification conditions were chosen so as to produce visible products by ethidium staining only for antibody-precipitated material, and not for "no antibody" controls. The fluorescent products were then run on an ABI PRISM 310 capillary sequencer (Applied Biosystems; Foster City, CA) to separate alleles differing in CAG repeat number and analyzed using GeneScan software (Applied Biosystems). Allele-specific expression analysis by RT-PCR was performed on DNaseI-treated RNA using a similar procedure [23].

Analysis of the 5' region of the *SYBL1 *gene was performed similarly to that of *AR*, except the allele-specific reaction entailed XhoI digestion of the primary amplification product followed by these products being separated by agarose gel electrophoresis. Conditions for PCR amplification and XhoI digestion were as previously described [31].

## List of abbreviations

ac = acetylated

ChIP = chromatin immune precipitation

DAPI = 4,6-diamidino-2-phenylidole

FB = fibroblast

FAM = 5-carboxyfluorescein

FITC = fluorescein-isothiocyanate

ICF = immune deficiency, centromeric region instability, facial anomalies

H3 = histone 3

H4 = histone 4

K4 = lysine 4

K9 = lysine 9

LINE-1 = long interspersed nuclear element 1

MeCP2 = methyl-CpG binding protein

WT = wild type

Xa = active X chromosome

Xi = inactive X chromosome

## Authors' contributions

SMG and RSH conceived the study design, supervised and coordinated its progress, and drafted and prepared the final manuscript. KRV and PL carried out the cell culture and cytological studies. TKC carried out the ChIP analyses. JT and UF developed the cloned Rett cell cultures. All authors read and approved the manuscript.

## Figures and Tables

**Table 1:** Inactive X histone modification patterns

Histone Modification	Normal	PT3 ICF	PT4 ICF	Rett 1	Rett 2
H3 acetylation^a^	-	-	-	-	-
H4 acetylation^a^	-	-	-	-	-
H3 methyl K9^b^	+	+	+	+	+
H3 methyl K4^a^	-	-	-	-	-
Macro H2A1^c^	+	+	+	+	+

^a^over 60% of DAPI-stained Barr bodies exhibit lack of antibody staining

^b^over 50% of DAPI-stained Barr bodies exhibit enhanced antibody staining

^c^over 80% of DAPI-stained Barr bodies exhibit enhanced antibody staining
